# Cancer screening and preventative care among long-term cancer survivors in the United Kingdom

**DOI:** 10.1038/sj.bjc.6605609

**Published:** 2010-03-16

**Authors:** N F Khan, L Carpenter, E Watson, P W Rose

**Affiliations:** 1 Department of Primary Health Care, University of Oxford, Oxford, UK; 2 Department of Public Health, University of Oxford, Oxford, UK; 3 School of Health and Social Care, Oxford Brookes University, Oxford, UK

**Keywords:** primary care, cancer screening, preventative care, cancer survivors

## Abstract

**Background:**

Long-term cancer survivors in the United Kingdom are mostly followed up in a primary care setting by their general practitioner; however, there is little research on the use of services. This study examines whether cancer survivors receive adequate screening and preventative care in UK primary care.

**Patients and methods:**

We identified a cohort of long-term survivors of breast, colorectal and prostate cancer with at least a 5-year survival using the General Practice Research Database, with controls matched for age, gender and practice. We compared adherence with cancer screening and the use of preventative care between cancer survivors and controls.

**Results:**

The cancer survivors’ cohort consisted of 18 612 breast, 5764 colorectal and 4868 prostate cancer survivors. Most cancer survivors receive cancer screening at the same levels as controls, except for breast cancer survivors who were less likely to receive a mammogram than controls (OR=0.78, 95% CI: 0.66–0.92). Long-term cancer survivors received comparable levels of influenza vaccinations and cholesterol tests, but breast (OR 0.81, 95% CI: 0.74–0.87) and prostate cancer survivors (OR=0.70, 95% CI: 0.57–0.87) were less likely to receive a blood pressure test. All survivors were more likely to receive bone densitometry.

**Conclusion:**

The provision and uptake of preventive care in a primary care setting in the United Kingdom is comparable between the survivors of three common cancers and those who have not had cancer. However, long-term breast cancer survivors in this cohort were less likely to receive a mammogram.

There are increasing numbers of cancer survivors in the United Kingdom. Current estimates indicate that there are now more than 2 million people living past a diagnosis of cancer in the United Kingdom, a number that is projected to increase by 3% annually (Maddams *et al*, 2009). With increasing numbers surviving many years after a diagnosis, it is important to identify and control adverse sequelae of cancer and its treatment, effectively manage comorbid conditions and optimise the health of this population (Aziz, 2002; Aziz and Rowland, 2003).

Cancer survivors are at increased risk of ill health because of a number of factors, including second cancers, late effects related to treatment and other comorbidities, including those unrelated to cancer (Curtis *et al*, 1985; Orel *et al*, 1993; Sankila *et al*, 1995). The risk of adverse health can be reduced through the use of preventative services that can have an important part in ensuring the long-term health of this population at risk (Carver *et al*, 2007). However, cancer survivors have previously been shown to report poorer health than the general population (Yabroff *et al*, 2004). Previous research into the use of preventative care provided to cancer survivors in the United States has provided mixed results. Although the use of preventive services was better in some cancer survivors, some breast cancer survivors had poorer uptake of measures such as cholesterol screening and influenza vaccination compared with non-cancer controls (Yeazel *et al*, 2004; Trask *et al*, 2005; Duffy *et al*, 2006; Snyder *et al*, 2009). In contrast, others found that although breast cancer survivors received adequate care, the uptake of preventative health in long-term colorectal cancer survivors was worse than in those without cancer (Earle *et al*, 2003; Earle and Neville, 2004). These disparities in health care in the United States may result partly from a focus on cancer follow-up at the expense of routine preventative care for other diseases.

Despite the long-term risks in this population, there has been very little research on the use of primary care services, and specifically on the cancer screening and preventative health behaviours, of cancer survivors in developed health economies other than the United States (Khan *et al*, 2008). This paper aims to fill this gap by examining the use of cancer and non-cancer-related preventative health practices in a primary care-based cohort of cancer survivors in the United Kingdom. In particular, we examined whether screening is affected by a previous cancer diagnosis, and whether cancer survivors receive similar preventative health care compared with individuals who have not had cancer.

## Methods

### The source of data

The General Practice Research Database (GPRD), which currently contains information on 3.6 million representative patients from 450 primary care practices in the United Kingdom, was used to identify the patients enrolled in this study (Walley and Mantgani, 1997). The GPRD includes records on individual-level clinical diagnoses, test results, prescriptions, referrals and all significant morbidity events in the patient's medical history (MHRA, 2004). The data undergo quality control procedures, and several validation studies have shown a high level of data completeness within the GPRD (Khan *et al*, 2010). Screening and preventative care events were identified if a medical or investigation code corresponding to the event was present in the patient medical record. Clinical and investigation code lists are available on request.

### Definition of preventative services

We examined adherence to three national cancer screening guidelines as outlined by the National Health Service (NHS) cancer screening programme and the use of services for routine screening of disease and disease prevention (Table 1). There are no British guidelines on frequency of blood pressure testing, bone density scanning or cholesterol tests; therefore, we examined the receipt of one test every 3 years.

### Description of participants

This study focuses on adult survivors of three cancer types: breast, colorectal and prostate cancer. We defined cancer survivors (cases) as those aged ⩾30 years at the time of diagnosis, with at least a 5-year survival after diagnosis. Controls were patients with no record of breast, colorectal or prostate cancer. For each case, up to four controls were selected from the same primary care practice and matched on the basis of age (within 1 year) and gender.

### Inclusion and exclusion criteria for the use of screening and preventative services

We only included patients who were within the age range and eligible for cancer screening, or who did not have a history of previous disease that would indicate the need for monitoring. We excluded women with a history of hysterectomy in the analysis of cervical smear, women with a history of bilateral mastectomy in the analysis of mammography, prostate cancer survivors in the PSA test analysis and breast cancer survivors <10 years after diagnosis in the use of mammography. For preventative services, we excluded patients with hypertension from blood pressure monitoring; patients with a clinical history of diabetes, cerebrovascular disease/stroke, hypertension and myocardial infarction from cholesterol testing; and patients with a clinical history of osteoporosis from bone density scanning. These patients will receive regular disease monitoring as directed by the Quality and Outcomes Framework, an incentive programme for payments to primary care practices from the English government (Department of Health, 2006).

### Statistical analyses

We compared the use of screening and preventative services between cancer survivors and their matched controls over a 3-year period between 1 September 2003 and 31 August 2006, which represented the most recent data available in our data set. We extended this analysis window to 5 years starting from 1 September 2001 for the analysis of cervical smear screening in women aged 50–64 years to capture events corresponding to the national cervical cancer screening programme guidelines (see Table 1 for screening recommendations). Cancer survivors entered the analysis along with their matched controls only when they achieved a 5-year survival. In addition, cancer survivors and their matched controls were censored from the analysis if the cancer survivor died, was diagnosed with a second cancer or was transferred out of the practice.

We first described the characteristics of the patients included in each analysis and examined the percentage of cancer survivors and controls receiving cancer screening and preventative services. To determine the association between cancer survivor status and use of preventative services compared with matched controls, we used conditional logistic regression to calculate odds ratios and 95% CIs, which accounts for the matching by comparing cases with the controls for whom they were selected (Kirkwood and Sterne, 2003). All analyses were carried out using Stata MP statistical software, version 10.1 (StataCorp LP, College Station, TX, USA).

### Explanatory variables

We assigned each patient a Charlson comorbidity score on the basis of their clinical history. The original Charlson score assigns patients with cancer a weighted score of 2; however, we excluded cancer as a comorbid disease when calculating individual Charlson scores for both cancer survivors and their controls (Charlson *et al*, 1987). We included the number of consultations during the study period as an explanatory variable for preventative care outcomes, as patients who consult more frequently are more likely to have incidental measurements, such as blood pressure. Information on mastectomy was particularly important when considering receipt of mammography. Therefore, we also linked the GPRD data set to treatment data obtained from the English national cancer registry network. A breast cancer patient was considered to have a history of bilateral mastectomy if there was a clinical record for bilateral mastectomy in GPRD records or cancer registration, or if there were two clinical codes for mastectomy more than 1 year apart. Not all mastectomy codes allowed us to distinguish between unilateral and bilateral mastectomy; hence, we included a history of mastectomy as a dichotomous covariate in the remainder of women without a specific code for bilateral mastectomy. We included a history of hormone therapy as identified through prescription records in the GPRD as a covariate in the analysis for bone density scanning. Body mass index was included in multivariate models that considered blood pressure, cholesterol testing and bone density scanning. As patients nearing the end of their life may be treated differently in primary care, we also included a dichotomous variable indicating whether the patient died during the analysis period, and tested for interactions between death and receipt of screening and preventative care.

## Results

### Patient characteristics and univariate analysis

The data set included 18 612 breast cancer survivors, 5764 colorectal cancer survivors, 4868 prostate cancer survivors and 116 418 controls (total *n*=145 662 patients). A summary of patient characteristics is shown in Table 2. Colorectal and prostate cancer survivors were quite elderly, and correspondingly a high proportion had at least one comorbid disease.

Table 3 shows the prevalence of screening and preventative service use among cancer survivors and controls. Rates of cervical smear were much lower than expected, but similar in all cancer survivors and controls, and a similar proportion of colorectal cancer survivors and controls underwent mammography according to the national guidelines. A lower proportion of breast cancer survivors underwent a mammogram compared with controls.

In general, cancer survivors were more likely to receive bone density scans, flu vaccination and cholesterol testing preventative care than the control population. However, breast cancer survivors were less likely to receive a blood pressure or cholesterol test compared with controls. We examined these differences in multivariate models.

### Cancer screening

After adjusting for a history of mastectomy (*n*=885), comorbid disease and death, breast cancer survivors were 22% less likely to receive a mammogram as indicated by the national screening programme, compared with their matched controls (Table 4). There was no difference in the receipt of mammography between female colorectal cancer survivors and matched controls. All breast cancer survivors were more likely than controls to have a cervical smear according to screening guidelines. There was no evidence for a difference in the utilisation of cervical smear among female colorectal cancer survivors compared with controls. As some women receiving mammography or cervical smear may have received screening just before or after the period of analysis, we conducted a sensitivity analysis allowing for a 3-month interval before and after the period of interest. The results were similar, and are therefore not presented.

Colorectal cancer survivors over the age of 50 years were 19% more likely to have a PSA test compared with controls. There was no evidence for a difference in screening behaviours among cancer survivors who died during the analysis period.

### Preventative care

A summary of the multivariate models that consider preventative care among cancer survivors and matched controls is presented in Table 4 (full multivariate models are included in Appendix 1). Each group of cancer survivors was more likely to receive bone density scanning than their matched controls. In addition, cancer survivors over the age of 65 years were more likely to receive an influenza vaccination. In contrast, there were no differences in cholesterol testing between cancer survivors and their matched controls. Although colorectal cancer survivors were as likely to receive a blood pressure test as their matched controls, both breast and prostate cancer survivors were less likely to receive a measurement of blood pressure over the analysis period. An increasing number of primary care visits was the strongest predictor of preventative care delivery.

## Discussion

This is the first paper reporting on the use of primary care services in a large population of cancer survivors outside the United States. In general, most cancer survivors in the United Kingdom receive similar cancer screening and preventative health compared with individuals who have not had cancer. All cancer survivors were more likely to receive influenza vaccination and bone density scans. However, in this cohort, long-term breast cancer survivors were less likely to receive mammography, and breast and prostate cancer survivors were less likely to receive a blood pressure test.

Second cancers can be detected at an earlier stage when appropriate screening guidelines are met (Adami *et al*, 2001). Colorectal cancer survivors in this cohort were more likely to receive a PSA test, and breast cancer survivors were more likely to receive a cervical smear than their matched controls. This finding is understandable; individuals who have previously had cancer may be motivated to identify second cancers at an early stage. However, it is of concern that long-term breast cancer survivors in this study are significantly less likely to receive breast cancer screening than women without a history of breast cancer. Breast cancer survivors are at risk of recurrence and primary cancer in the contralateral breast; therefore, even women who have a unilateral mastectomy should receive regular mammography (Burstein and Winer, 2000). This is the not the first paper to report the underuse of breast cancer screening; previous research has shown the underuse of mammography in breast cancer survivors and childhood cancer survivors despite an increased risk of mortality (Andersen and Urban, 1998; Schapira *et al*, 2000; Keating *et al*, 2006; Lash *et al*, 2007; Oeffinger *et al*, 2009). Although lack of access to mammographic services contributes to the underuse of breast screening amongst certain populations in the United States, this is unlikely to explain cancer screening practices in the United Kingdom, as all eligible women are invited for free breast cancer screening through a national programme. It is difficult to explore the reasons behind attendance at national cancer screening programmes in a large, anonymised database study. However, to increase screening rates, it is important to understand why the use of mammography is lower in long-term survivors.

The results from this analysis indicate that that in UK primary care, cancer survivors generally receive similar preventative care compared with matched controls, and indeed in some cases, receive better care. It seems that the bone health of cancer survivors is well managed in the United Kingdom; our analysis shows that breast and prostate cancer survivors, who are at a greater risk of osteoporosis after hormonal treatment, are significantly more likely to receive a bone density scan than matched controls (Chen *et al*, 2005). This is a large study with a greater propensity for significant results, and although there are deficiencies in the receipt of blood pressure testing among breast and prostate survivors, the differences are small and may not be clinically significant.

It is important to consider the results from this UK-based paper and previous US-based studies in the context of differences in national health-care delivery. In the United States, patients who consulted their oncologist only in the long-term received poorer preventative care for other diseases, but those who consulted both primary and secondary care physicians received the best care (Earle *et al*, 2003; Earle and Neville, 2004; Snyder *et al*, 2008, 2009). Earle and colleagues suggested that a lack of clarity of the roles of secondary and primary care in the United States may lead to fragmentation of care, with cancer survivors unclear about whom to consult for different aspects of their health care. Long-term cancer survivors in the United Kingdom are unlikely to maintain contact with oncologists, and will rely on their GP to provide care for their cancer and other comorbidities after discharge from hospital follow-up. Compared with the United States, the role of primary care in the general health care of cancer survivors in the United Kingdom is well defined, and this difference may explain why cancer survivors in this cohort received preventative care at similar levels to a non-cancer population. With the exception of mammography for breast cancer survivors, there is certainly no suggestion that cancer survivors are penalised by their status.

Several potential limitations of this study should be considered. First, the data source was collected primarily for clinical use rather than for research, and it is possible that some tests and diagnoses were not fully recorded. However, because the analysis was conducted as comparisons between cases and controls, completeness of recording should not affect this analysis; it is unlikely that the services considered in this paper would be coded preferentially in either population. One proxy of the completeness of recording in the GPRD is a comparison of rates of screening and preventative care with national data. Rates of mammography through the national screening programme are currently 73%, and hence may be slightly underestimated in this GPRD cohort (2009). However, rates of influenza captured in the GPRD closely mirror those of national influenza vaccination, as estimated by the Department of Health (NHS Immunization Information, 2005). This difference in completeness of recording may reflect the fact that influenza vaccination is likely to take place in primary care; however, breast cancer screening occurs in specialised screening centres, and GPs may record the event differently in the GPRD electronic patient record. However, cervical screening, which does occur in UK primary care, was not well captured in this GPRD cohort. National coverage of cervical screening is ∼78% therefore, the rates of cervical smear are drastically underestimated in this study (2008). This underestimate is a recognised problem in the GPRD because of underrecording of cervical smear results in computerised patient medical records before the widespread use of electronic transfer of cervical smear results from laboratories.

Second, it has been suggested that research using administrative data may underestimate rates of bilateral mastectomy (Nissen *et al*, 2008). However, the analysis in this paper was conducted using surgery data from both cancer registries and primary care. Some of the clinical codes used to identify mastectomy in the GPRD were not precise enough to determine whether the procedure was bilateral or unilateral. This may be a limitation, as patients receiving a bilateral mastectomy will not be eligible for breast screening. However, to account for this, we excluded any women with a specific primary care code for bilateral mastectomy, or two mastectomy codes more than a year apart. This definition of bilateral mastectomy resulted in an overall rate of 4.2% in the cohort of breast cancer survivors, which corresponds to rates in the SEER (Surveillance, Epidemiology and End Results) database (Tuttle *et al*, 2007). We also conducted a sensitivity analysis excluding all women with any clinical code for mastectomy. The results were similar and therefore not presented.

Finally, because we are using a primary care database, we are unlikely to account for preventative care or screening occurring in hospital or in specialised clinics. However, in the United Kingdom, the majority of cancer survivors are discharged to the care of their GP 3 to 5 years after diagnosis. Therefore, the majority of their care will occur in primary care and is likely to be coded in the GPRD.

As cancer survivors are living longer, the current challenge in primary care is to maximise the quality and duration of life after cancer. This can be achieved through screening, preventative services and advice to patients, which can reduce the risk of second cancers and other potential comorbidities. There are now guidelines for risk-based surveillance among childhood cancer survivors; management of bone loss among breast cancer survivors; and identification of cardiac and pulmonary late-effects in long-term survivors; however, more work is required in this area to direct GPs who will be responsible for the long-term health care of this population (Carver *et al*, 2007; Reid *et al*, 2008; Hudson *et al*, 2009). There is a need for evidence-based guidelines to direct case finding and preventative care in primary care for elderly cancer survivors, who require care of general health and comorbid disease, as well as potential late effects of cancer and its treatment. A further understanding of the factors that contribute to late effects and the appropriate risk-based surveillance will help towards maintaining the good health of this population.

## Figures and Tables

**Table 1:** Summary of UK guidance for cancer screening and preventative health services

	**National guidance**	**Age range (years)**	**Frequency of test**
*Screening test*			
Mammogram	NHS Breast Cancer Screening Programme (2007–2008)	50–69	Once every 3 years
Cervical smear	NHS Cervical Cancer Screening Programme (2007–2008)	25–49	Once every 3 years
		50–64	Once every 5 years
PSA test	Prostate Cancer Risk Management Programme (PCRMP) (UK National Screening Committee, 2001)	50+	Patient request
			
*Preventative health*			
Influenza vaccination	National Institute for Health and Clinical Excellence (NICE) (2008c)	65+	Annually
Blood pressure test	None	—	—
Bone density scan	None	—	—
Cholesterol test (HDL, LDL, serum cholesterol)	None	—	—

Abbreviations: HDL=high-density lipoprotein; LDL=low-density lipoprotein; PSA=prostate-specific antigen.

**Table 2:** Patient demographics

	**Breast**		**Colorectal**		**Prostate**	
	**Survivor**	**Control**	**Survivor**	**Control**	**Survivor**	**Control**
*Numbers in analysis*						
Male	—	—	2912	11 515	4868	19 288
Female	18 612	74 284	2852	11 331	—	—
						
Age in years (s.d.)	67.65 (12.65)		75.13 (11.2)		76.86 (8.31)	
Charlson score >0	8594 (46.2%)	31384 (42.3%)	3418 (59.3%)	12022 (52.6%)	3189 (65.5%)	11008 (57.1%)

**Table 3:** Univariate analysis, with numbers eligible for analysis

	**Breast**		**Colorectal**		**Prostate**	
	**Survivor**	**Control**	**Survivor**	**Control**	**Survivor**	**Control**
*Screening exam*						
Mammogram	54.4%	59.5%	60.5%	58.5%		
Number in analysis	2317	9974	513	2055	—	—
Cervical smear						
Aged 25–49 years	43.6%	40.1%	13.9%	15.2%	—	—
Number in analysis	755	3085	65	263		
Aged 50–64 years	45.7%	44.4%	41.6%	41.9%	—	—
Number in analysis	3419	14 061	178	855		
PSA test	—	—	22.1%	19.0%	—	—
Number in analysis			2489	9859		
						
*Preventative health*						
Influenza vaccination	75.8%	74.4%	74.9%	73.2%	74.6%	70.2%
Number in analysis	9488	37 803	4118	16 325	3854	15 275
Blood pressure	68.0%	69.2%	70.1%	70.4%	70.7%	68.7%
Number in analysis	9771	38 815	2531	10 668	2105	9252
Bone density scan	14.1%	10.3%	13.2%	10.5%	17.1%	9.3%
Number in analysis	14 574	59 471	4455	17 954	3928	16 026
Cholesterol test	31.9%	33.1%	37.8%	36.2%	39.9%	36.7%
Number in analysis	9072	35311	2306	9235	1720	7563

Abbreviation: PSA=prostate-specific antigen.

**Table 4:** Multivariate model for receipt of screening and preventative care (odds ratios from conditional logistic models)

	**Breast cancer**	**Colorectal cancer**	**Prostate cancer**
*Screening* a			
Mammogram	0.78, 95% CI 0.66–0.92	1.11, 95% CI 0.77–1.61	—
Cervical smear			—
Aged 25–49 years	1.37, 95% CI 1.11–1.68	0.66, 95% CI 0.21–2.15	—
Aged 50–64 years	1.14, 95% CI 1.03–1.25	1.13, 95% CI 0.72–1.75	—
PSA testing		1.19, 95% CI 1.06–1.34	—
			
*Preventative care*			
Influenza vaccination^b^	1.15, 95% CI 1.07–1.23	1.15, 95% CI 1.03–1.28	1.39, 95% CI 1.22–1.59
Blood pressure test^c^	0.81, 95% CI 0.74–0.87	0.97, 95% CI 0.81–1.17	0.70, 95% CI 0.57–0.87
Bone density scan^c^	1.26, 95% CI 1.10–1.44	1.23, 95% CI 1.05–1.43	1.59, 95% CI 1.23–2.06
Cholesterol testing^c^	0.93, 95% CI 0.87–1.00	1.01, 95% CI 0.85–1.18	0.83, 95% CI 0.69–1.01

Abbreviations: BMI=body mass index; CI=confidence interval; PSA=prostate-specific antigen.

Cancer controls were used as the baseline comparison.

a Adjusted for Charlson comorbidity score and death. Mammography analysis also adjusted for history of mastectomy.

b Adjusted for total number of consultations over analysis period, Charlson comorbidity score and death.

c Adjusted for total number of consultations over analysis period, BMI, Charlson score and death. Bone density scan analysis also adjusted for hormone treatment in breast and prostate cancer.
